# Image Quantification for TSPO PET with a Novel Image-Derived Input Function Method

**DOI:** 10.3390/diagnostics12051161

**Published:** 2022-05-07

**Authors:** Yu-Hua Dean Fang, Jonathan E. McConathy, Talene A. Yacoubian, Yue Zhang, Richard E. Kennedy, David G. Standaert

**Affiliations:** 1 Department of Radiology, Heersink School of Medicine, University of Alabama at Birmingham, Birmingham, AL 35294, USA; jmcconathy@uabmc.edu; 2 Center for Neurodegeneration and Experimental Therapeutics, Department of Neurology, Heersink School of Medicine, University of Alabama at Birmingham, Birmingham, AL 35294, USA; tyacoubian@uabmc.edu (T.A.Y.); dstandaert@uabmc.edu (D.G.S.); 3 Department of Medicine, Heersink School of Medicine, University of Alabama at Birmingham, Birmingham, AL 35294, USA; yuezhang@uabmc.edu (Y.Z.); richardkennedy@uabmc.edu (R.E.K.)

**Keywords:** TSPO PET, neuroinflammation, image-derived input function, kinetic modeling analysis

## Abstract

There is a growing interest in using ^18^F-DPA-714 PET to study neuroinflammation and microglial activation through imaging the 18-kDa translocator protein (TSPO). Although quantification of ^18^F-DPA-714 binding can be achieved through kinetic modeling analysis with an arterial input function (AIF) measured with blood sampling procedures, the invasiveness of such procedures has been an obstacle for wide application. To address these challenges, we developed an image-derived input function (IDIF) that noninvasively estimates the arterial input function from the images acquired for ^18^F-DPA-714 quantification. Methods: The method entails three fully automatic steps to extract the IDIF, including a segmentation of voxels with highest likelihood of being the arterial blood over the carotid artery, a model-based matrix factorization to extract the arterial blood signal, and a scaling optimization procedure to scale the extracted arterial blood signal into the activity concentration unit. Two cohorts of human subjects were used to evaluate the extracted IDIF. In the first cohort of five subjects, arterial blood sampling was performed, and the calculated IDIF was validated against the measured AIF through the comparison of distribution volumes from AIF (V_T,AIF_) and IDIF (V_T,IDIF_). In the second cohort, PET studies from twenty-eight healthy controls without arterial blood sampling were used to compare V_T,IDIF_ with V_T,REF_ measured using a reference region-based analysis to evaluate whether it can distinguish high-affinity (HAB) and mixed-affinity (MAB) binders. Results: In the arterial blood-sampling cohort, V_T_ derived from IDIF was found to be an accurate surrogate of the V_T_ from AIF. The bias of V_T, IDIF_ was −5.8 ± 7.8% when compared to V_T,AIF_, and the linear mixed effect model showed a high correlation between V_T,AIF_ and V_T, IDIF_ (*p* < 0.001). In the nonblood-sampling cohort, V_T, IDIF_ showed a significance difference between the HAB and MAB healthy controls. V_T, IDIF_ and standard uptake values (SUV) showed superior results in distinguishing HAB from MAB subjects than V_T,REF_. Conclusions: A novel IDIF method for ^18^F-DPA-714 PET quantification was developed and evaluated in this study. This IDIF provides a noninvasive alternative measurement of V_T_ to quantify the TSPO binding of ^18^F-DPA-714 in the human brain through dynamic PET scans.

## 1. Introduction

In recent years, the role of neuroinflammation has been studied in many neurodegenerative diseases, including Alzheimer’s disease (AD) and Parkinson’s disease (PD) [1,2,3,4]. Noninvasive measurement of regional brain microglial activation with PET imaging has become a popular approach for investigation of neuroinflammation in clinical research [5]. The best established and most often used PET imaging biomarker for microglial activation is the 18 kDa translocator protein (TSPO), a protein abundant in brain microglia, monocytes, and other macrophages. A variety of studies have shown the usefulness of the ^18^F-DPA-714 in research studies of microglial involvement in neurological disorders [6,7]. Despite the growing usage of ^18^F-DPA-714 in clinical research, quantification of the ^18^F-DPA-714 uptake and binding to TPSO remains a challenge for clinical research studies. Conventionally, ^18^F-DPA-714 binding is quantified through a kinetic modeling analysis, where the compartmental analysis is conducted over the tissue time-activity curves from the dynamic PET studies. However, the main obstacle for such analysis is the invasive nature of the required arterial blood sampling procedure to acquire the arterial input function (AIF). Processing of the arterial blood samples is a time-consuming and complex procedure that affects the feasibility to include AIF measurement in clinical trials.

To avoid the arterial blood sampling procedures, modeling approaches based on reference regions are commonly adopted to quantify cerebral tracer binding for PET imaging. To use a reference region, there is an underlying modeling assumption that the reference region is devoid of specific binding of the PET tracer. However, in the case of ^18^F-DPA-714, there is a widespread distribution of TSPO in the normal brain, and no region can be regarded as a perfect reference region lacking TSPO binding, especially if microglial activation is widespread. For example, the cerebellum has been a popular reference region of choice in the literature for TSPO binding quantification [8,9,10,11]. However, it has been well known that the cerebellum contains a substantial amount of specific binding sites for TSPO tracers [12,13]. Previous reports have suggested that in such cases, reference region methods may lead to a biased measurement of tracer binding [14]. Moreover, the binding capacity in the reference region may be altered under pathological or pharmacological conditions. For example, Gerhard et al. showed that the cerebellum shows elevated TSPO overexpression with the TSPO tracer ^11^C-(R)PK11195 in subjects with progressive supranuclear palsy [15]. Increased TSPO tracer binding in the cerebellum has also been observed in AD [16,17]. In some conditions, there could even be a global elevation of neuroinflammation and TSPO overexpression throughout the brain [18]. Under such cases, it is nearly impossible to identify a reference region that can properly serve as a true reference region. As a result, quantifying TSPO PET with reference region methods may not be an appropriate choice, especially if a disease or abnormality may cause widespread TSPO overexpression and microglial activation throughout the brain.

To address these limitations, we sought to develop an image-derived input function (IDIF) as a noninvasive surrogate for the AIF measurement. Current methods for extracting IDIF are usually based on image segmentation techniques that focus on extracting large arterial structures such as carotid arteries [19,20] or the left ventricle [21]. Due to the relatively low spatial resolution of PET, such methods need to address the activity spillover and partial volume effects that lead to a mixture of the true blood activity and activity from surrounding tissue [19,22] or use a few blood samples to correct for the activity cross-contamination [20]. An IDIF extraction method for mouse TSPO PET imaging has been developed based on factor analysis by Wimberley et al. [23], but it requires a whole body scan and may be difficult for human brain PET studies without specialized scanners. Currently there does not seem to be a satisfactory IDIF solution for human TSPO PET imaging.

In this work, we developed a new IDIF method by using a model-based matrix factorization (MBMF) to separate the arterial blood and brain tissue radioactivity. We also developed a unique optimization procedure to scale the extracted IDIF from a normalized and dimensionless form into the activity concentration of the arterial blood signal. The developed method was validated through two approaches. First, we validated our method in a small cohort (*n* = 5) in which we conducted arterial blood sampling and measured AIF directly. The calculated IDIF was compared with the measured AIF through a Logan graphical analysis that measures the volume of distribution (V_T_). Second, we applied the IDIF method to a group of healthy controls (*n* = 28), which had been genotyped for the polymorphism (rs6971) that determines affinity for ^18^F-DPA-714 for TSPO [24]. Subjects predicted to be high-affinity binders (HAB; rs6971 C/C) and mixed-affinity binders (MAB; rs6971 C/T) were included in the cohort, while low-affinity binders (LAB, rs6971 T/T) were not included in the imaging study. Previous investigations have shown a 20–50% higher V_T_ for ^18^F-DPA-714 in HAB subjects compared to MAB subjects [12,25]. We evaluated whether V_T_ measured with IDIF was able to detect these expected differences in binding in our cohort of healthy controls. Standard uptake values (SUVs) from the 40th to the 60th minutes post injection were also taken as an alternative reference of comparison between the HAB and MAB groups.

## 2. Materials and Methods

### 2.1. Standard Protocol Approvals, Registrations, and Patient Consents

Two separate cohorts of human subjects were recruited for this study. For cohort 1, all subjects were recruited under a small pilot study to examine the utility of ^18^F-DPA-714 imaging for studying neuroinflammation in PD (ClinicalTrials.gov Identifier: NCT03457493). For cohort 2, human subjects were recruited as part of the larger longitudinal NINDS-funded Alabama Udall Center observational study examining the role of inflammation in early PD. Participants were enrolled between March 2018 and May 2021 through the Movement Disorder Clinic at the University of Alabama at Birmingham. The study was approved by institutional review board at UAB, and full written consent was obtained on each participant.

### 2.2. Participants

For cohort 1, denoted as the blood-sampling cohort in this work, eligible participants were age ≥30 years and were healthy controls or subjects diagnosed with PD. Control subjects had no current diagnosis of PD or other neurodegenerative disorder, had no history of PD in first-degrees blood relatives, and had ≤3 positive response on the PD Screening Questionnaire [26]. Subjects with PD were diagnosed according to the United Kingdom Brain Bank criteria by a movement disorder specialist. These criteria require bradykinesia and at least one of the following: 4–6 Hz resting tremor, rigidity, or postural instability. There was no restriction to stage of PD for enrollment.

For cohort 2, denoted as the nonblood-sampling cohort, imaging data from MAB and HAB control subjects enrolled as part of the larger longitudinal NINDS-funded Alabama Udall Center study prior to May 2021 were used for the analysis. Eligible participants were age ≥40 years. Control subjects had no current diagnosis of PD or other neurodegenerative disorder, had no history of PD in first-degrees blood relatives, and had ≤3 positive response on the PD Screening Questionnaire [26]. Subjects were excluded if they had a history of significant autoimmune/inflammatory disorder, current treatment with immunosuppressant therapy, or serious comorbidity that would interfere with study participation.

All participants underwent genetic testing for the rs6971 SNP associated with TSPO binding and were classified into low-, mixed-, or high-affinity binders. Low-affinity binders were not imaged.

### 2.3. Data Acquisition for the Cohort with Arterial Blood Sampling Procedures

In the blood-sampling cohort, five subjects underwent arterial blood sampling procedures during their ^18^F-DPA-714 PET scans. These five subjects included two healthy controls and three PD patients. The TSPO genotype was determined by measuring the rs6971 polymorphism of the TSPO gene with single-nucleotide polymorphism (SNP)-based tests. Three of them were HAB, and two were MAB. Arterial blood samples were collected through the radial artery catheter under the following sampling settings: one sample per six seconds for the first minute, one sample per ten seconds for one minute, one sample per minute for three minutes, and one sample every five minutes for the rest of the scan, yielding a total of thirty samples for each study. Both the whole-blood and plasma activity concentrations of ^18^F-DPA-714 were measured from each arterial blood sample. The blood samples at the 5th, 15th, 30th, and 60th minutes post injection were analyzed with high-performance liquid chromatography (HPLC) to measure the parent fraction for unmetabolized ^18^F-DPA-714 for four subjects of the study. Decay correction was performed for all blood samples. All subjects underwent dynamic ^18^F-DPA-714 PET/MR scans. Injection dose for ^18^F-DPA-714 was 5 mCi (185 MBq). The ^18^F-DPA-714 PET scan with a GE Signa PET/MR scanner lasted for 60 min for each subject immediately after tracer injection. Images were reconstructed with OSEM using 4 iterations and 16 subsets into a total of 36 frames, with the frame setting of 12 ten-second, 9 20-second, 5 one-minute, and 10 five-minute frames. Attenuation correction was involved in the reconstruction process with MR-based attenuation maps acquired with zero echo time (ZTE) MRI [27]. Time-of-flight information and point spread functions were incorporated in the PET reconstruction. The image volume was 256 × 256 × 89 with the pixel size of 1.17 mm and a slice thickness of 2.78 mm. Decay correction was also performed during the PET reconstruction.

### 2.4. Data Acquisition for the Cohort without Arterial Blood Sampling

The nonblood-sampling cohort included only healthy controls who underwent PET scans but did not undergo arterial blood sampling during the PET acquisition. Fifteen subjects out of the 28 healthy controls were HAB, and thirteen subjects were MAB. All subjects underwent the same PET dynamic acquisition as described previously.

### 2.5. Image Post-Processing

The reconstructed dynamic PET data underwent two additional image correction processes. First, we conducted a frame-by-frame 3D PET image registration to minimize the between-frame misalignment due to the involuntary patient motion during the PET acquisition. The last frame was used as the reference for the frame-by-frame registration. Second, partial volume correction was performed for all PET datasets with the geometric transfer matrix method [28] provided by the PETPVC toolbox [29]. The anatomical maps were derived from the segmentation results using Freesurfer, which performs subcortical region segmentation over the T1-weighted scans [30]. All image processing methods were implemented in MATLAB (version 2020a, Mathworks, Inc., Natick, MA, USA) The Freesurfer-derived segmentation maps of the prior step were also applied to the PET dynamic data and used to extract the tissue time-activity curves of the regions of interest. We chose the following nine regions as target regions of evaluation: putamen, caudate, thalamus, hippocampus, frontal cortex, temporal cortex, occipital cortex, parietal cortex, and cerebellum.

### 2.6. IDIF Extraction Procedure

The IDIF extraction procedure involved three steps: an image segmentation process to extract arterial voxels, a factorization process to separate the blood signal from the tissue signal, and a scaling process to set the separated blood signal back to the accurate activity concentration units. In the first step, we aimed to segment the voxels that are most likely to be within the carotid arteries. For each subject, we first took the Freesurfer-derived segmentation maps to determine the lowest slice of the cerebellum and only segment between this slice and the overall lowest slice in the field of view. For each of those axial slices, we took the first minute of dynamic frames and searched on the left side for the voxel with the highest intensity over these six frames. Assuming an approximated diameter of 6 mm for the carotid arteries [31], this voxel was then dilated with a five-by-five diamond-shaped structural element to form a segmented mask. The same operation was repeated for the right side of the same image slice to complete the carotid segmentation for this slice. All the selected slices underwent the same segmentation procedure to complete the segmentation for carotid arterial blood voxels. With the partial volume effect, the intensity of the segmented voxels that resemble the arterial blood activities is in fact a mixture of the arterial blood activities and the surrounding tissues.

To extract the arterial blood activities, it is assumed that all the surrounding tissue of the selected voxels can be approximated as a single tissue type and shares the same tracer uptake kinetics. Accordingly, the activity concentration of a specific voxel *i* can be expressed as:

(1) $$C_{i,PET}\left(t\right)=\alpha_{i}C_{AIF}\left(t\right)\;+(1-\alpha_{i})C_{TISSUE}\left(t\right)$$

where $C_{PET}$ represents the PET-measured activity, $C_{AIF}$ represents the arterial blood activity, and $C_{TISSUE}$ represents the surrounding tissue activity. $\alpha_{i}$ is the voxel-dependent mixing fraction of the arterial blood for the specific voxel.

Assuming there is a total of *n* segmented voxels and m PET image frames for the dynamic study, an n-by-m matrix A can be formed by combining $C_{i,PET}$ from all the m frames and n segmented voxels. With the goal to extract the underlying arterial input function $C_{AIF}$ and tissue activity $C_{TISSUE}$, we developed a novel method that decomposes the matrix A into a 2-by-m matrix H that contains the two components of $C_{AIF}$ and $C_{TISSUE}$ and an n-by-2 weighting matrix W so that the difference between A and W*H can be minimized. Unlike other blind matrix factorization methods, our matrix factorization method is based on physiological models and shares similar concepts with guided matrix factorization [32] and knowledge-driven matrix factorization [33] by incorporating the prior knowledge of underlying factors during the matrix factorization process. During this model-based matrix factorization (MBMF), it is assumed that $C_{AIF}$ and $C_{TISSUE}$ can both be modeled and parameterized. Accordingly, the factorization process becomes an optimization problem that estimates the underlying parameters for $C_{AIF}$ and $C_{TISSUE}$, instead of a blind and direct search for the time activity curves of $C_{AIF}$ and $C_{TISSUE}$. In this study, we used the 7-parameter input function model developed by Feng et al. [22,34] as:

(2) $$C_{AIF}\left(t\right)=\left(A_{1}(t-\tau \right)-A_{2}-A_{3})e^{-\lambda_{1}\left(t-\tau \right)}+A_{2}e^{-\lambda_{2}\left(t-\tau \right)}+A_{3}e^{-\lambda_{3}\left(t-\tau \right)}$$

And the tissue time-activity function $C_{TISSUE}$ was modeled as a two-tissue compartment model output as [35]:

(3) $$C_{TISSUE}\left(t\right)=C_{AIF}\left(t\right)⊗\frac{K_{1}}{\left(B_{2}-B_{1}\right)}\left[\left(k_{3}+k_{4}-B_{1}\right)e^{-B_{1}t}+\left(B_{2}-k_{3}-k_{4}\right)e^{-B_{2}t}\right]$$

where

(4) $$B_{1,2}=\frac{1}{2}\left[(k_{2}+k_{3}+k_{4}\right)\mp \sqrt{{\left(k_{2}+k_{3}+k_{4}\right)}^{2}-4k_{2}k_{4}}]$$

We used the trust-region-reflective optimization algorithm within MATLAB’s ‘fmincon’ function to perform the numerical optimization for the $C_{AIF}\left(t\right)$ and $C_{TISSUE}\left(t\right)$. In each iteration of the parameter optimization, the parameter set of $\emptyset =\{\tau ,A_{1},A_{2},A_{3},\lambda_{1},\lambda_{2},\lambda_{3},K_{1},k_{2},k_{3},k_{4}$} at the current iteration was applied to Equations (2) and (3) and then used to form matrix H. The weighting matrix W at this iteration was derived from MATLAB’s linear system solver that minimizes the least-square errors. The optimization then searched for the optimal solution for the parameter set ∅ by minimizing:

(5) $$\hat{\emptyset }=\arg\min_{\left(\emptyset \right)}{\left||WH-A|\right|}^{2}$$

After the convergence of MBMF, optimal ∅ was used to determine the extracted and normalized functions $C_{Norm,AIF}$ and $C_{Norm,TISSUE}$ that represented the decomposed arterial blood and tissue components, respectively. Those functions were denoted with ‘Norm’ because they were in a normalized form after the MBMF extraction, where $\overset{m}{\underset{j=1}{\sum }}C_{Norm,AIF,j}$ equaled to one (*j* is the frame index). The last step was to scale the extracted $C_{Norm,AIF}$ from an arbitrary unit to the correct physical magnitude of activity units as $C_{AIF}$. To scale $C_{Norm,AIF}$ to the correct units of activity concentration, we related $C_{Norm,AIF}$ to the true AIF $C_{AIF}$ by a scaling factor $s_{AIF}$ as:

(6) $$C_{AIF}\left(t\right)=s_{AIF}C_{Norm,AIF}\left(t\right)$$

Similarly, $C_{Norm,TISSUE}$ was related to the true $C_{TISSUE}$ by a scaling factor $s_{TISSUE}$. The individual activity of a specific voxel *i* was estimated by the MBMF extraction as:

(7) $${\tilde{C}}_{i,PET}\left(t\right)=w_{i,AIF}C_{Norm,AIF}\left(t\right)+w_{i,TISSUE}C_{Norm,TISSUE}\left(t\right)$$

$w_{i,AIF}$ and $w_{i,TISSUE}$ were the MBMF-estimated mixing fractions for the $C_{Norm,AIF}$ and $C_{Norm,TISSUE}$, respectively. Note ${\tilde{C}}_{i,PET}$ is not identical to $C_{i,PET}$ since it is a weighted summation of the extracted and estimated functions $C_{Norm,AIF}$ and $C_{Norm,TISSUE}$.

We further derived:

(8) $${\tilde{C}}_{i,PET}\left(t\right)=\frac{\omega_{i,AIF}}{s_{AIF}}\cdot C_{AIF}\left(t\right)+\frac{\omega_{i,TISSUE}}{s_{TISSUE}}\cdot C_{TISSUE}\left(t\right)$$

From Equation (1), it was assumed that the mixing fractions of the AIF and tissue activity should sum up to one under ideal situation. Therefore, the optimal values of $s_{AIF}$ and $s_{TISSUE}$ were optimized by minimizing:

(9) $${\hat{s}}_{AIF},\;{\hat{s}}_{TISSUE}=\arg\min_{\left(s_{AIF},\;s_{TISSUE}\right)}\overset{n}{\underset{i=1}{\sum }}{\left(\frac{\omega_{i,AIF}}{s_{AIF}}+\frac{\omega_{i,TISSUE}}{s_{TISSUE}}-1\right)}^{2}$$

where ${\hat{s}}_{AIF}$ and ${\hat{s}}_{TISSUE}$ denoted the optimized scaling factors for extracted AIF and tissue activity, respectively. n denoted the total number of segmented voxels. We used the trust-region-reflective optimization algorithm with MATLAB’s ‘fmincon’ function to perform the numerical optimization for minimizing Equation (5). The estimated ${\hat{s}}_{AIF}$ was then plugged into Equation (6) to scale the IDIF into the correct physical units.

### 2.7. Metabolite Correction

Since the IDIF method extracts the whole-blood activity and cannot separate the metabolite signal from the unmetabolized tracer, we used a population-based approach to convert the extracted IDIF to a metabolite-corrected plasma time-activity curve [36,37]. With the metabolite data and plasma time-activity curves measured in the blood-sampling cohort, the individual parent fraction was multiplied to the plasma-to-whole blood activity fraction. The individual composite fraction was averaged for each time point at the 5th, 15th, 30th, and 60th minutes and then fitted to a single exponential function with constant [37,38]. As a result, the corrected IDIF is expressed as:

(10) $$C_{IDIF,MCPC}\left(t\right)={\hat{s}}_{AIF}C_{N,AIF}\left(t\right)(1-0.29(1-e^{-0.03t}))$$

where *t* is in the unit of minutes, and *MCPC* denotes metabolite-corrected plasma concentration of activity.

### 2.8. ^18^F-DPA-714 Quantification

Quantification of ^18^F-DPA-714 binding was estimated through the Logan plot, where the distribution volume (V_T_) was approximated by the graphical analysis [39,40]. Logan plot analysis was based on the 30th to the 60th minutes of data [41]. For the blood-sampling cohort, the distribution volume V_T_ was calculated from the metabolite-corrected AIF, denoted as V_T,AIF_. The distribution volume was also calculated from the metabolite-corrected IDIF, denoted as V_T,IDIF_ and compared to V_T,AIF_. For each subject, nine target regions were chosen from the Freesurfer segmentation and calculated for V_T,AIF_ and V_T,IDIF_. For the nonblood-sampling cohort, V_T,IDIF_ was calculated with the same steps described. The distribution volume with respect to the reference region, denoted as V_T,REF_, was also computed with the cerebellum time-activity curves using the Logan graphical analysis.

### 2.9. Statistical Analysis

In the blood-sampling cohort, V_T,IDIF_ was compared against the reference V_T,AIF_ with data presented as mean ± SD. Error percentage, presented as mean ± SD, was calculated for V_T,IDIF_ using V_T,AIF_ as the gold standard value. Linear mixed-effect model was used to examine the correlation between V_T,IDIF_ and V_T,AIF_, treating the subject as a random effect with or without the region as a fixed effect.

In the nonblood-sampling cohort, the subjects were divided into the HAB and MAB groups based on the individual genotypes of TSPO binding. The V_T,IDIF_ of the HAB group was compared to that of the MAB group through an unpaired two-sample *t*-test. Significance was set as *p* < 0.05. The same analysis was also performed for the SUV as well as V_T,REF_ to evaluate their differences between the HAB and MAB groups. Linear mixed effect model was used to evaluate whether there was a significant difference between the HAB and MAB under SUV, V_T,IDIF_, and V_T,REF_, respectively, adjusting for the effects of region with subject as a random effect.

## 3. Results

### 3.1. IDIF Predicts AIF

**Figure 1:**
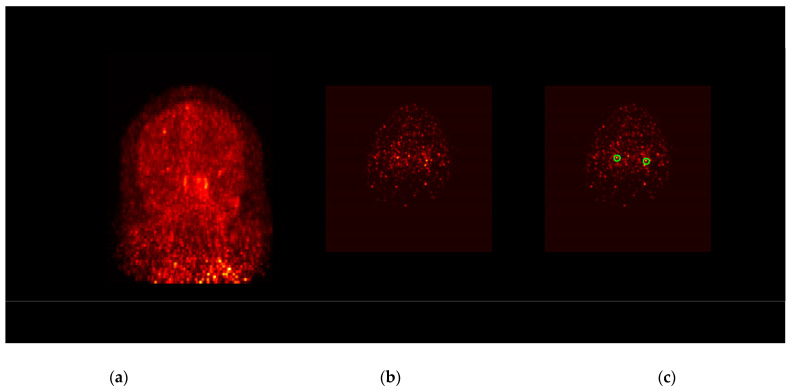

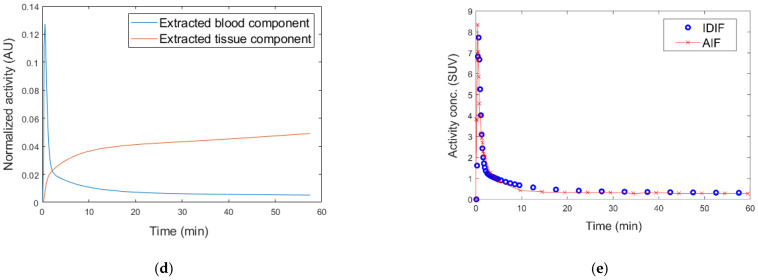
Demonstration of the IDIF extraction. (**a**) The coronal maximum intensity projection (MIP) of the summed early frames of the DPA-714 scan of one subject. (**b**) One axial slice of the neck area at the early frame. (**c**) The segmented regions (green contour) for carotid artery over the same slice. (**d**) The MBMF-extracted time-activity curves for the blood and tissue components. Note both curves are in the normalized and dimensionless form. (**e**) The resultant IDIF (blue circle) after the magnitude is re-scaled by the proposed method. The activities measured with arterial blood sampling are plotted as red stars, showing a satisfactory agreement between AIF and IDIF.

In the first cohort of five subjects, arterial blood sampling was performed to measure the AIF. We then compared the distribution volume calculated with the AIF (V_T,AIF_) and IDIF (V_T,IDIF_) to determine whether IDIF is an appropriate surrogate for measurement of ^18^F-DPA-714 quantification. The extraction process of the IDIF in one subject of the blood-sampling cohort is demonstrated in Figure 1 for the three steps: segmentation, signal decomposition, and scale optimization. Figure 1a shows the summed PET image over the first 60 s post tracer injection as a maximum intensity projection. Figure 1b,c show one axial slice within the neck area and the segmented contour for the carotid arteries. The extracted tissue and blood components are shown in Figure 1d, and the IDIF after the scale adjustment is plotted in Figure 1e with the measured arterial input function (AIF). The IDIF curve demonstrated a satisfactory agreement with the AIF curve. $\frac{\omega_{i,AIF}}{s_{AIF}}$ averaged 0.28 ± 0.30, and $\frac{\omega_{i,TISSUE}}{s_{TISSUE}}$ averaged 0.55 ± 0.36 across the five subjects. The comparison of IDIF and AIF for the other four subjects in the blood-sampling cohort is shown in Figure S1.

**Figure 2:**
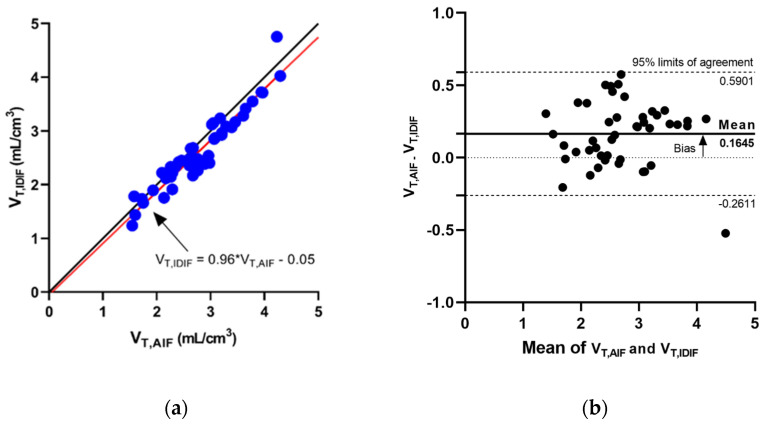
(**a**) The scatter plot for V_T,IDIF_ plotted against V_T,AIF_. The dashed line is the unity line. The red line is the fitted regression trend line. The scatter plot shows a strong correlation between V_T,IDIF_ plotted against V_T,AIF_. The statistical test also showed a strong correlation through the mixed effect model analysis. (**b**) The Bland–Altman plot for V_T,IDIF_ and V_T,AIF_.

Table 1 summarizes the V_T_ derived from the AIF and IDIF for the nine target regions in the blood-sampling cohort. The overall error for V_T,IDIF_ was −5.8 ± 7.8% against the reference V_T,AIF_. In all regions, the mean error of V_T_ was less than 10% across all subjects. The amount of error for V_T_ in the healthy controls is similar in the PD patients. Figure 2 shows the scatter plot and the Bland–Altman plot for V_T,IDIF_ and V_T,AIF_ of all the target regions and demonstrates a satisfactory agreement between them. In the linear mixed effect model analysis, the overall V_T,IDIF_ and V_T,AIF_ were highly correlated with each other (*p* < 0.001), adjusting for the effect of regions.

### 3.2. IDIF Method Distinguishes High-Affinity Binders from Mixed-Affinity Binders

**Figure 3:**
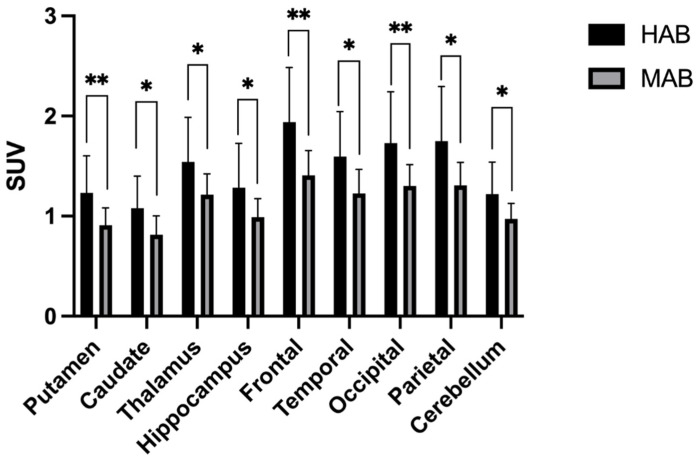
The box plot of the SUV measured for target brain regions for high- and mixed-affinity binders in the nonblood-sampling cohort. * denotes significant differences under two-sample *t*-test (*p* < 0.05), while ** denotes *p* < 0.01. All nine target regions showed significant differences between the HAB and MAB subjects. SUV is 31 ± 3% higher in HAB than in MAB.

**Figure 4:**
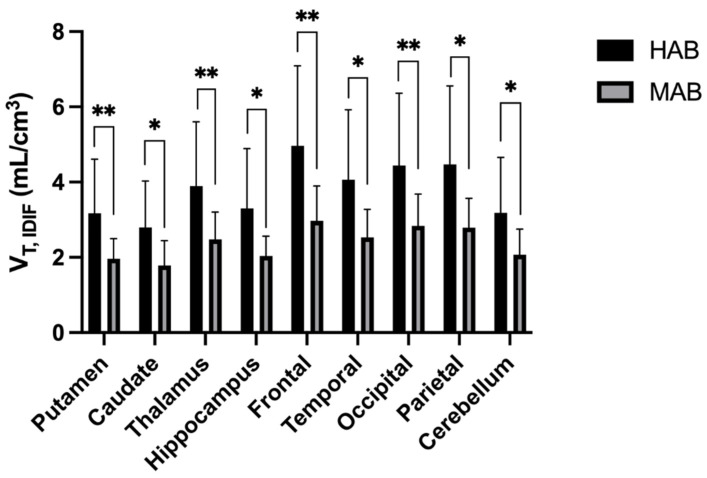
The box plot of the V_T,IDIF_. V_T,IDIF_ is 37 ± 3% higher in HAB than in MAB. All nine regions were found with significant difference (*: *p* < 0.05, **: *p* < 0.01) between the HAB and MAB subjects. The overall increase pattern in the HAB is similar as the SUV pattern of increase.

**Figure 5:**
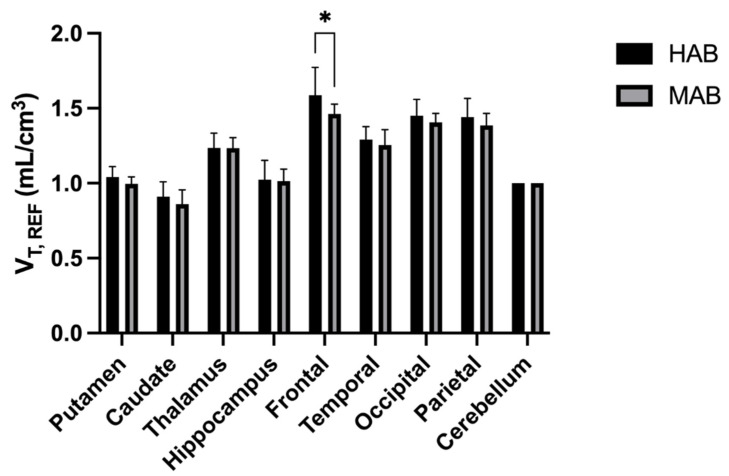
The box plot of the V^T,REF^ using the cerebellum as the reference region. The difference between HAB and MAB is 3 ± 3% and only reaches significance (*: *p* < 0.05) in just one of nine regions.

To validate our IDIF quantification methodology, we next tested whether IDIF could distinguish control subjects who were MAB from those who were HAB as determined by TSPO SNP genotyping. In the SUV measurements, all nine brain regions showed significantly higher uptake of ^18^F-DPA-714 in the HAB group than in the MAB group (*p* < 0.05). The HAB uptake averaged 31 ± 3% higher than the MAB in SUV (Figure 3). The distribution volume as determined by IDIF also showed significantly higher uptake in HAB vs. MAB overall (*p* < 0.05). Region-wise, mean V_T,IDIF_ was statistically higher in the HAB group in all nine brain regions (Figure 4). V_T,IDIF_ was 37 ± 3% higher in the HAB group overall. On the other hand, the distribution volume determined by using the cerebellum as a reference region showed only a mildly increased V_T,REF_ of 3 ± 3% in the HAB group compared to the MAB group (Figure 5). Only one out of the nine brain regions revealed a statistically significant increase in V_T,REF_ in HAB vs. MAB subjects with the reference region approach. The mixed effect model showed a significant difference in the SUV (*p* = 0.010) and V_T,IDIF_ (*p* = 0.010) but not in the V_T,REF_ (*p* = 0.069) after adjusting for the effects of regions.

## 4. Discussion

Evidence is growing for the central role of neuroinflammation in many neurodegenerative diseases, and accordingly neuroinflammation is both a potential marker for diagnosis and a therapeutic target. As a tool of noninvasive measurement of neuroinflammation, TSPO PET has gained much interest in recent year but poses unique challenges in quantification. Interested readers are referred to a comprehensive review of TSPO PET quantification by Wimberley et al. [42]. One challenge that has been recognized for ^18^F-DPA-714 imaging is how to achieve an accurate quantification of the tracer binding through kinetic modeling analysis. Since microglial cells are widely distributed in all brain tissues and neuroinflammation can potentially occur throughout the brain, the underlying assumption for reference region-based analysis may be violated when a certain disease affects the reference region and increases the microglial activation similarly to the target region of interest. Although the cerebellum has been used as the reference region by several reports, it is also well known that the cerebellum demonstrates non-negligible ^18^F-DPA-714-specific binding even in the healthy subjects [12,13]. Therefore, it is not surprising that prior reports have shown that the cerebellar TSPO binding can be elevated in certain diseases that make the cerebellum further deviate from the modeling assumptions for the reference regions [15,16,17]. Studies have suggested that several neurological disorders can cause globally elevated neuroinflammation including the cerebellum [18,43,44]. For example, Terada et al. have shown that there may be a global pattern of microglial activation in the whole brain for PD patients [45]. Under such a scenario, the reference region-based method may fail to properly measure the ^18^F-DPA-714 binding differences between the study groups. Accordingly, an accurate and non-invasive method for DPA-714 quantification is significant for TSPO PET in measuring neuroinflammation.

Here, we have shown that our developed IDIF method is a potential alternative for arterial blood sampling methods. This method incorporates image segmentation, signal separation, and a novel approach to scale the extracted TACs to the accurate magnitude. Based on a relatively small validation cohort, our current results show a satisfactory extraction of IDIF that was very similar to the AIF calculated from arterial blood sampling. We found that the V_T_ measured with AIF and IDIF is highly correlated (*p* < 0.001), and the difference between these two measurements is small with less than 10% overall bias. The data from this cohort show that the IDIF-measured V_T_ decently resembles the AIF-measured V_T_ and may be a useful alternative to replace the V_T_ measured through invasive arterial blood sampling. The proposed method is fully automatic and may eliminate the potential interoperator variabilities. The fact that it does not require any blood sampling or sample processing makes this approach easy to apply to retrospective data analysis and to use in clinical trials. Further studies with larger validation cohorts would be crucial for a more comprehensive validation and performance evaluation for this method.

We further validated the IDIF method by testing whether IDIF can distinguish HAB vs. MAB healthy control subjects. TSPO genotypes, specifically a single nucleotide polymorphism at rs6971, critically affect the ^18^F-DPA-714 signal. Prior reports have shown that the TSPO ligand binding in HAB subjects is 20–50% higher than that of MAB subjects, depending on the quantification approaches and study settings [12,25]. In our dataset, a simple measurement of SUV shows significantly higher ^18^F-DPA-714 uptake in the HAB group that matched the expected magnitude of increase as described in the literature. When IDIF-based kinetic modeling analysis was applied, similar results were obtained as the degree of V_T,IDIF_ increase was similar to the SUV increase. All of the nine tested regions showed significant differences through V_T,IDIF_ as expected. On the other hand, the V_T_ measured using reference region-based analysis with the cerebellum as the reference region showed only a minimal increase in the HAB group of less than five percent. Only one out of nine brain regions showed significant differences between the HAB and MAB subjects using reference region-based analysis. This lack of difference is likely an artifact of the reference region method, arising from the fact that TSPO binding is increased in both the target and reference regions for the HAB group compared to the MAB group. Similar results have been presented in a study conducted by Hameline et al. in which the cerebellum was chosen as the reference region. In this study, the ^18^F-DPA-714 SUVr obtained from the HAB and MAB subjects was very similar using the reference region-based analysis, supporting our observation that the cerebellum may not serve as an ideal reference region for TSPO imaging as it may cancel out or diminish the effects of TSPO overexpression caused by certain physiological or pathological conditions [46]. Our data suggest that the developed IDIF method may be more suitable for quantifying the TSPO binding than reference region methods.

Other efforts have been developed to noninvasively extract the input function or reference region activities for kinetic modeling analysis. An approach similar to IDIF is the population-based input function (PBIF) method [37]. This method assumes an identical curve shape for arterial input functions across the population. A PBIF can be obtained by averaging the AIF for a cohort with the individual scale determined through one or few blood samples. Compared with the PBIF method, the IDIF method developed in this work estimates the individual curve shape for AIF and scales the estimated AIF with the imaging data. No blood sampling or population AIF data are required in our approach, and therefore it may be easier to apply the IDIF over dynamic PET scans. The supervised clustering algorithm (SVCA), on the other hand, extracts the voxels that most closely follow the tracer kinetics of a low-binding, time-activity curve that is predefined from previously collected cohort datasets [47]. SVCA methods are fully automatic and have been applied in the image quantification of several disease models [48]. However, the challenge for SVCA is that a predefined set of kinetic curves must be present and known for both the healthy controls and subjects with the specific brain disorder that is being studied. In addition, such predefined kinetic curves must be scanner- and protocol-specific, and such requirements may limit its applicability for analyzing the data acquired through clinical trials where the patient sample sizes are often limited [42]. Moreover, some reports have also suggested that the SVCA-extracted reference region time-activity curves may still contain a non-negligible amount of specific binding that may lead to bias in quantifying the TSPO binding [49,50]. It requires further studies to objectively compare the performances of the proposed IDIF method with SVCA and PBIF methods to determine which may provide the most reliable quantification of TSPO binding, and it may likely be dependent on the disease model being investigated.

This study has its limitations, and the proposed method can be further improved. First, our blood-sampling cohort contained only five subjects due to the difficulties of performing arterial blood sampling procedures, particularly under the influences of the global SARS-CoV-2 pandemic during the subject recruitment. A larger cohort with blood sampling may help further verify the accuracy and reliability of the developed IDIF method. Second, since our method is based on matrix factorization to extract the IDIF, the accuracy of the extracted IDIF will depend on the segmented voxels that ideally shall be those possessing high fractions of the arterial blood. Our current segmentation method is a simple method that searches for voxels that are likely to be within the carotid artery. Although it has the benefit that it does not require data from modalities other than PET, the carotid segmentation can certainly be improved with more advanced methods or with the assistance of MR- or CT-based angiography. For example, some of the IDIF methods make use of time-of-flight MR angiography (TOF-MRA) through a simultaneous PET/MR to delineate the carotid arteries [51]. The enhanced segmentation of arterial structures may provide the MBMF with a better source data for matrix factorization and therefore improve the accuracy of IDIF extraction. Third, our experimental design included a 60 min PET dynamic acquisition to reduce the discomfort for the recruited patients, whereas a 90 min acquisition has been more common in the current literature. Although prior reports have shown that a 60 min scan may properly suffice for an accurate measurement of V_T_ [12,41], a longer scan would be beneficial to increase the parameter sensitivity toward the estimation of binding potential and microrate constants through compartment modeling analysis. Although our proposed IDIF method is not strictly dependent on the scan protocol, how our method would perform under a longer scan requires future studies to evaluate. Fourth, since the IDIF can only extract whole-blood AIF, individual metabolite and plasma activity correction will not be feasible without additional blood sampling procedures. Accordingly, a population-based approach for metabolite correction was taken in this work. Whether the error of V_T_ measurement is introduced by such a population-based method requires further investigation. Lastly, signal separation of the IDIF and tissue tracer uptake could possibly be improved in our method with other signal separation methods, such as those based on machine learning techniques [52].

## 5. Conclusions

A novel image-derived input function method for quantifying the TSPO binding with ^18^F-DPA-714 was developed in this work. We used two separate cohorts as an initial validation for this method and showed that it may serve as a promising alternative for an automatic and noninvasive way to extract the IDIF.

## Figures and Tables

**Table 1:** Comparison between the V_T,AIF_ and V_T,IDIF_ in the blood-sampling cohort. Overall error is −5.8 ± 7.8% for V_T,IDIF_ when compared to V_T,AIF_. The error does not appear to be dependent on the target region.

	All (*n* = 5)			PD (*n* = 3)			HC (*n* = 2)		
	V_T,AIF_	V_T,IDIF_	Error %	V_T,AIF_	V_T,IDIF_	Error %	V_T,AIF_	V_T,IDIF_	Error %
Putamen	2.44 ± 0.52	2.30 ± 0.52	−5.7 ± 7.7	2.61 ± 0.47	2.43 ± 0.59	−7.7 ± 9.7	2.19 ± 0.95	2.12 ± 0.79	−2.7 ± 5.4
Caudate	1.91 ± 0.48	1.81 ± 0.50	−5.4 ± 11.9	1.91 ± 0.60	1.83 ± 0.61	−3.8 ± 16.4	1.93 ± 0.70	1.79 ± 0.73	−7.6 ± 3.8
Thalamus	2.97 ± 0.63	2.77 ± 0.61	−6.7 ± 7.0	3.12 ± 0.71	2.89 ± 0.75	−7.6 ± 9.4	2.75 ± 1.17	2.59 ± 1.06	−5.3 ± 1.8
Hippocampus	2.61 ± 0.58	2.42 ± 0.44	−6.3 ± 9.3	2.79 ± 0.62	2.57 ± 0.45	−6.6 ± 12.6	2.34 ± 0.96	2.19 ± 0.84	−6.0 ± 3.0
Frontal	2.70 ± 0.49	2.55 ± 0.48	−5.1 ± 7.5	2.79 ± 0.46	2.65 ± 0.54	−5.2 ± 9.5	2.56 ± 0.88	2.41 ± 0.77	−5.0 ± 3.2
Temporal	3.45 ± 0.86	3.34 ± 1.08	−4.5 ± 11.5	3.73 ± 0.92	3.65 ± 1.33	−4.2 ± 15.7	3.03 ± 1.29	2.86 ± 1.12	−4.8 ± 2.8
Occipital	2.96 ± 0.55	2.78 ± 0.55	−6.1 ± 7.3	3.15 ± 0.45	2.95 ± 0.57	−6.5 ± 9.9	2.69 ± 1.05	2.52 ± 0.92	−5.5 ± 4.8
Parietal	3.13 ± 0.52	2.97 ± 0.47	−5.0 ± 7.0	3.26 ± 0.45	3.08 ± 0.51	−5.7 ± 8.7	2.93 ± 0.97	2.79 ± 0.80	−3.9 ± 4.1
Cerebellum	3.16 ± 0.60	2.95 ± 0.58	−6.4 ± 7.1	3.35 ± 0.55	3.11 ± 0.67	−7.4 ± 9.7	2.87 ± 1.14	2.70 ± 0.97	−5.0 ± 1.5

## Data Availability

The data presented in this study are available on request from the corresponding author. The data are not publicly available due to the privacy of the enrolled subjects.

## Supplementary Materials

The following supporting information can be downloaded at: https://www.mdpi.com/article/10.3390/diagnostics12051161/s1, Figure S1: The comparison between AIF and IDIF for the other four subjects in the blood-sampling cohort.
