# Supplementary material for: Image Quantification for TSPO PET with a Novel Image-Derived Input Function Method
Source: Diagnostics (Basel). 2022 May 7;12(5):1161. doi: 10.3390/diagnostics12051161 (PMC9140104; doi:10.3390/diagnostics12051161)
Supplement: Supplementary file 1 [file diagnostics-12-01161-s001.zip › diagnostics-1681089-supplementary.pdf]

Supplemental data

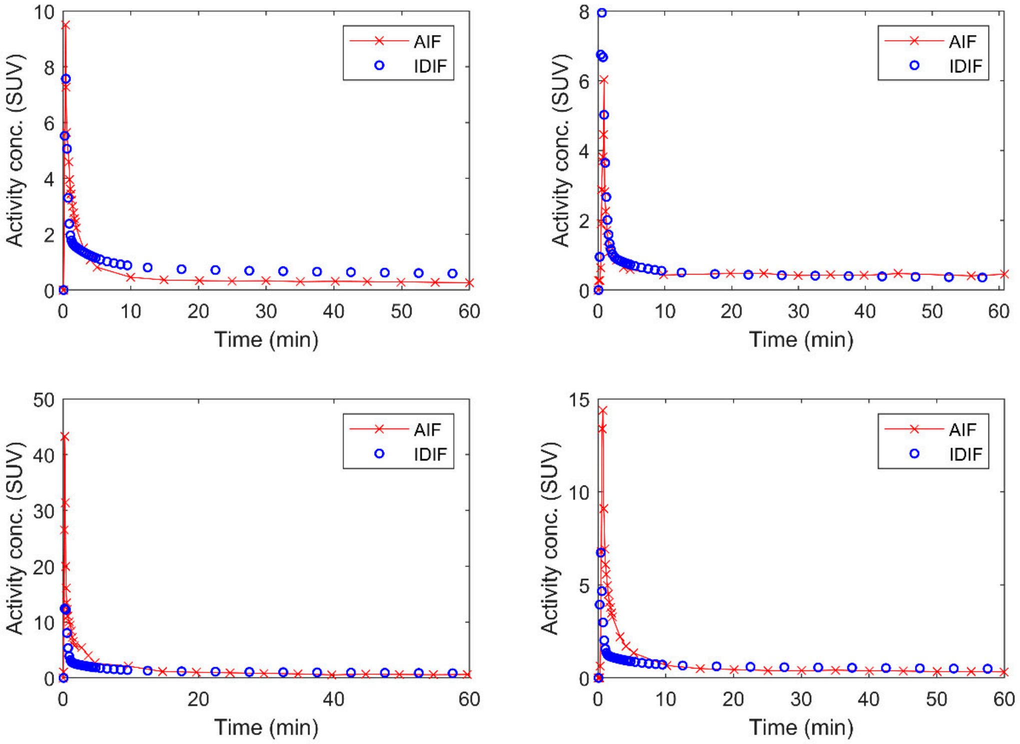

Supplemental Figure S1. The comparison between AIF and IDIF for the other four subjects in the blood-sampling cohort.
